# Proceedings of the Maryland and District of Columbia Dental Society

**Published:** 1879-12

**Authors:** 


AMERICAN JOURNAL

OF

DENTAL SCIENCE.

Vol. XIII. THIRD SERIES—DECEMBER, 1879. No. 8.

### ARTICLE I.

[Continued from last Number.]

*Proceedings of the Maryland and District of Columbia Dental Society. Annual Meeting.*

EVENING SESSION, 8 P. M.

The Association was called to order by the President.

On motion the rules were suspended, and Dr. James McPherson being nominated, was duly elected.

The reorganization of Sections being the special order of business, the following named gentlemen were elected Chairmen:

Section 1—Dr. R. B. Winder.

" 2 " W. H. Atkinson.

" 3 " E. P. Keech.

" 4 " J. J. Caldwell.

" 5 " C. E. Duck.

" 6 " R. Finley Hunt.

" 7 " H. C. Thompson.

" 8 " B. F. Coy.

" 9 " W. W. Evans.

" 10 " R. B. Donaldson.

On motion, the numbers of the Sections were changed so that they should come in order, viz:

Number 1—Operative Dentistry.
" 2—Metallurgy and Chemistry of Metals.
" 3—Artificial Dentistry.
" 4—Dental Education.
" 5—Anatomy and Physiology.
" 6—Histology and Microscopy.
" 7—Pathology and Etiology.
" 8—Diagnosis and Therapeutics.
" 9—Chemistry and Materia Medica.
" 10—Anæsthesia and Anæsthetics.

The President called for reports of Sections.

Dr. Thompson stated that for the first time since he had been a member of the Association he was ashamed to say he had no report. He communicated with each member of his section and received no answer. He proposed this year to make the members of his section work.

Dr. Coy, Chairman of section on Operative Dentistry, read the following report:

We will refer to, (in the form of queries,)—

1st: The relative value of the different preparations of gold for filling and restoring lost parts of teeth, also contour, *versus* simple flush fillings. When should self-cleansing spaces be left between teeth, and when contoured to close at the grinding surface, or supra-contoured at the cervical wall to prevent lodgment of food at the necks of teeth, (bicuspids and molars,) the gold to extend a little above the free margin of the gum and flush to the border thereof. This contour can form a longitudinal ridge approaching an edge that will prevent any lodgment that may force the gums further from the necks of teeth. 2nd query: Under 20 years of age, what is the best material or materials for *saving* frail, badly decayed front teeth? Shall leave important points open for discussion of these suggestions. One week would not afford time to make a full report.

2nd: We might ask in this place what are the necessary qualifications for a dental surgeon, but will say in a few words what no one will take exception too, viz. that he should possess a minute knowledge of the anatomy of the oral cavity, both as to its soft and hard parts, together with their relations to and with other parts of the organism —this cavity as a cause of disease and its symptoms of disturbance in remote parts by reflex irritation.

His knowledge of physiology should be general and thorough, and at least a sound knowledge of dental therapeutics. This comes more properly under section 10, but this is where the two Sections meet and are interwoven.

3rd: In diagnosis of the pathological conditions of this cavity, (by this I mean that decay of the teeth is a pathological condition, and here again we find ourselves slipping into Section 4,) the dental surgeon must know what health is in order to understand the departures from it. Whether or not the teeth are in a condition (and the surrounding tissues,) to operate upon at once and without treatment? How much and what treatment will be necessary before an operation can be ventured upon? Also in operations upon the dental organs, he must appreciate the relations of the teeth and mouth to remote parts of the organism. For example, would he be justified in subjecting a patient, pregnant, to a severe operation in filling or the still more injurious operation of extracting teeth, unless allowing the tooth to remain would be the worst of the two alternatives. Among the tumors and other growths of the oral cavity he must be able to distinguish between the malignant and non-malignant; between the cyst and the pus secreting formations, and all the different diseases, symptomatic and otherwise, that present themselves on the free margin of the gums and the peridental membranes.

4th: If he does not know what constitutes a regular set of teeth—a proper occlusion—his attempts to regulate misplaced organs, or a crowded condition of the arches, will prove often, a worse than failure—facial distortion. If he is wise, he will not attempt to do what others claim to have done, when careful study of the case will show the result sought for hardly compatible with art and science, and interference not justifiable. Narrow arches contracted—often hereditary—will not hold a full set of large teeth.

5th: What was once the province of the dentist "to extract teeth and stop holes and make false teeth," is now happily elevated to the higher plane of science and art, and in demanding of the dental practitioner to protect these organs from decay and other modes of destruction, and the removal of teeth *required* in the arches a *denier resort*, after all other means for their restoration have been exhausted. The present standing of our profession, with all the modern improvements at our behest, including the different materials for arresting decay and restoring form to organs that have lost parts or whole of their crowns; we have but one more step, it would seem, to have all we could desire as operative dentists, and that would be a material sufficiently durable, and the color desired, and not affected by thermal changes—adhesive to the walls of the cavity formed and capable of resisting any and all acids of the oral cavity, whether to the manor born or introduced from without, then our glittering operations in that god of metals would be replaced by less show, but more worth (to the patient,) and less artificial dentures.

6th: Mr. President, had I not been compelled to attempt to do the work for my entire section, I intended to undertake a paper on dental surgery proper of the oral cavity and adjacent parts. We are all doubtless familiar with Dr. Garretson's operation of removing the entire tonsil in acute tonsilitis or quinsy, by the steel wire with temper drawn, after twisting the tonsil from between the two curtains of muscular anterior and posterior by the turning of a bent needle, thus avoiding the danger of the knife to the internal carotid, and no bleeding from annoyance. Will refer to only one operation, that the removal of the *epulis tumor*. My own practice has been to remove with the scalpel the tumor from its attachment to the alveolus and neck of the tooth or teeth, but *not* extract the teeth, which has heretofore been insisted upon, to prevent its recurrence. I take a large burr (in the engine,) and burr all the alveolus entirely off that has been covered by the tumor, and even the peridental membranes. Another operation would be to extract and scrape only that portion of the tooth where tumor connected with membrane of the tooth socket, and replace the teeth.

7th: I trust that hereafter the Chairman of this section will not have to toil alone. It is an important one; the health of the speaker has been bad, and I suggest that the combinations of this section with sections 4 and 8, might be well.

The above report is not what I know the Association is entitled to, but such as it is, is most respectfully submitted.

The President announced the subject open for discussion.

Dr. Hunt. Briefly I would say with reference to the paper just read, that it touches upon a point which is a subject of very earnest discussion in our profession — that is the *manner* in which proximate cavities should be treated. One fact seems to me to be left out of the calculation in these discussions, and that is that the teeth that require filling, in spite of the utmost after pains taken with them, are very liable to decay in other places than those that have been filled. For instance: a tooth decays on the proximal surface, the cavity is properly prepared and properly filled; yet you still have a portion of the tooth between the filling and the gum, in which food can lodge and remain in contact with the tooth, producing a recurrence of the trouble. It seems to me this point should have more attention than it receives. I had occasion to fill some such teeth for a patient within the last ten days, in which in the mesial proximal surface of a second bicuspid, the filling had been apparently well put in. But decay had recurred between the gold and the original wall of the cavity, and I found not only decay there, but the whole surface of the tooth between this buccal edge of the filling and the gum was partially disintegrated. Now this recurrence of decay would have occurred undoubtedly whether the filling had been contoured or flush. The teeth were not widely separated. The decay was caused by decomposing food, the remedy I must confess I am not able to point out except in general terms. We must direct our attention to the constitutional condition of the patient and remove any vice that might exist. If that tooth had been filled, and what is called a self-cleansing space left between the two, I do not think it would have decayed in that position. While we are directing our attention to the method of inserting a filling, and to the form, whether flush or contour, we should also direct our attention particularly to the cause of the original decay in that mouth, and guard against that cause in future, and also in other parts of the mouth.

I want to maintain two points that have not been expressed in operative dentistry. One is that gold of itself produces no electrical action. The second is of more importance since the promulgation of the utterances and positions of the new departure corps. That is while amalgam fillings conform to the general forms of the cavities, the surfaces do not, but are rough and indented, thus being liable to leak. In view of this I want every one to make specimens with amalgam, and I think if they will fill a cavity either in a tooth or in a hole made for the purpose, and break it open and examine with a magnifying-glass, they will see that it is not so smooth a surface as would prevent fluids from entering between the filling and the walls of the cavity. I have been surprised to find so much difficulty in producing between the surface of the cavity and the amalgam, the smooth regular surface there should be. Until we do that, amalgam will not fit the cavities as gold fillings do.

Right in that connection we are fain to agree to some extent with the gentlemen of the new-departure when they, through Dr. Flagg, take the position that in some instances it is probably better for the tooth that the filling does leak. What a state of affairs is that placed before us? We see fillings of amalgam that have been in for five, ten or twenty years that are preserving the tooth, but the tooth is black. Why is this so? We must follow out our investigations until we say certainly what it is. Foster Flagg is not understood by his professional brethren. I do not think it will do for any one to condemn him until he has examined the matter thoroughly, and finds ground for condemnation. Our own experience has been that amalgam does preserve teeth that gold would not.

Dr. Foster. Mr. President, I would like to ask Dr. Hunt if in his experiments he found the amalgam to contract or expand?

Dr. Hunt. I will say I have made no experiments in reference to expansion or contraction. My experiments have been as to the surfaces.

Dr. Foster. What is the doctor's belief as to the expansion or contraction in mixing?

Dr. Hunt. I can only give my belief without any ground for it. Do you mean after its insertion or while setting? I must say I have not been able to make up my mind, whether it is expansion or contraction.

Dr. Volck. Mr. President, some years ago there was a great deal said about using amalgam among the older operators. We are cautioned against amalgam. They made us promise at the commencement never to use amalgam.

I made myself some blocks of steel and had holes drilled through them. The blocks were about a half-inch thick. The holes were carefully drilled through, ranging in size from a sixteenth to a quarter of an inch. I filled the holes in one block with the greatest care with the best amalgams I could get; the corresponding block with gold. The amalgam was put in with as little mercury as possible. After a few days it was very hard and everything seemed tight. You could take the block filled with gold and you would have to use a heavy hammer to drive the plugs through, but in every instance I found with very little pressure I could push my amalgam through. The gold was polished when it came through, but the amalgam was rough. It proved to me that there was an absolute contraction in the setting of the amalgam.

Dr. Foster. That is not exactly what I want to get at. Everyone I think believes that is the case. But the question is in the mixing, whether the contraction takes place at that time or afterward. In the experiment the gold being softer, might spread under the hammer and go through harder than the amalgam, it being harder and less liable to spread.

Dr. Hunt. I want to say here that this new-departure corps is composed of seven gentlemen. They are divided into sections. One has charge of the mechanical, another of the metallurgical, and a third of the dental part. The result of their experiments with reference to amalgams, agree with Doctors Volck and Foster, that amalgams do shrink. After a certain length of time the edges of the amalgam is seen above the tooth.

Dr. Foster. That fact shows that there has been expansion at the same time.

Dr. Hunt. I will state for the information of the gentlemen present, that the ground taken by these gentlemen who have experimented is that this projection, if the filling has been in the mouth for a length of time, is caused by an effort of the amalgam to assume a spheroidal shape. They say that mercury always does this; every little piece assumes a spheroidal shape. In assuming this shape it presses against the sides of the cavity and very naturally presses out of the only opening it has. I think that every gentleman of the profession living in Baltimore have seen at Snowden & Cowman's, a tube filled by Flagg. The surface of that material is beautiful as the surface of the silvering of our mirrors. The silvering of our mirrors is done by having the amalgam so wet with mercury as to fit the back of the mirror perfectly. This filling does fit the surface of the tube perfectly, but you can not get out of all the amalgams one that will produce that same effect under your hands. I have tried them all. I have seen Dr. Winder fill a tooth in the hand and break it open to show me; and there is apparently a smooth surface on that portion of the amalgam in contact with the tooth, but not such a surface as would prevent leakage. If we can make amalgam so that we can place it in the cavity and have the surfaces of it fit the cavity; come in contact with the dentine as we can our gold fillings, then we will have no more black fillings. We may have the surface black, but the surfaces in contact with the tooth will not discolor.

In my paper I stated that filling materials containing chlorine cannot be relied upon for permanent fillings. The grounds stated there are sufficient to account for the failure of plastic fillings made with other materials than alloys. And the fact of one, two, or occasional fillings lasting in the mouth for a number of years, does not neutralize the force of the grounds taken in that conclusion. It only shows in my opinion that in those cases where these fillings have lasted a long time, there were no acidulous agents to act upon those fillings. I have placed plastic fillings in different parts of the same mouth. I have found some of them last well and others not so well. I have tested those mouths and found the fluids gave a different reaction. I think every one will bear me out when I say plastic fillings made of oxcyhloride of zinc, or phosphate of zinc or those other salts, are not to be relied upon as lasting any length of time when placed near the gum. I have placed these oxychloride fillings in such cavities and found they did not last, and that portion of the filling nearest the gum would fail first, and fail entirely before the other parts would be effected. A piece of red litmus paper placed under the free edge of the gum, will, in four cases out of five show an alkaline reaction. I was much interested in a case that came to me recently: I extracted a cuspidatus which could not be saved. The bicuspid next to it was getting into the same condition. The lower bicuspids had a very large amount of salivary calculus upon them. I tested the secretions under the free border of the gums in this case, and I found that the red litmus paper became immediately blue; in some parts of the mouth a deeper blue than would be produced by the fumes of ammonia.

Dr. Duck. In reference to plastic fillings, I must say my experience has been just the reverse of Dr. Hunt's. I have found oxychloride fillings frequently last upon the proximate surfaces of first and second molars, better than in most other localities. I have seen them there for a great many years, and found them in a good state of preservation, and I attribute the success in these particular localities to the slight deposit of carbonate of lime, coming from the saliva at these points. They seem to be coated with this carbonate of lime, and that seems to keep the fillings intact.

Dr. Hunt. I do not pretend to say Mr. President, that this is so in all cases, but these cases form the exceptions rather than the rule, and simply show that these agents have not been at work. If any one finding these fillings lasting a long time will test the secretions of that part of the mouth, he will find them very nearly neutral in their action. I think if Dr. Duck will look back, he will find that comparatively speaking, the number of plastic fillings that are preserving the teeth for any length of time is very small.

Dr. Duck. Yes sir, I believe it is so.

Dr. Winder. Dr. Coy asks in the commencement of his paper a very important question. That is, whether teeth should be contoured under the age of twenty, or should be separated. This is a question which I think involves everything. It is deep seated; reaches a long way. I believe the form of the tooth has more to do with its resistance to decay than almost anything else; certainly more than anything else, admitting the tooth substance to be of equal quality.

Dr. Coy. I would like to correct Dr. Winder. I asked the question, what is the best material under twenty years of age for preserving teeth? I did not ask anything about cutting teeth away.

Dr. Winder. We have this practice of cutting teeth carried to the bitter end by a gentleman of this city; that is Dr. Arthur. I think he went so far at one time as to lay down the bold proposition that if any of the incisor teeth were decayed on their proximate surfaces before twelve years of age, then all the other teeth should be separated by cutting apart. There are others who believe in contouring under any and all circumstances. I appeal to all of you gentlemen to know if you can get teeth up to the typical form, do they decay at all? If you take the teeth of an Indian or a Negro, or the finest white race, where they come up to your ideal of perfect formation, do they decay at all? I have found that this was certainly so in the examination of the teeth of the crania of the savage races. Observe this formation. You will notice that these typical or ideal teeth have single points of contact, and that this point is near the cutting edge. Having only one single point of contact with no broad surfaces in opposition, food does not pass in readily, and even should it do so it at once passes into an open, self-cleansing space. With such teeth decay is exceedingly rare. I have noticed further that where the round, regular arch is found, that but little crossing of races has occurred. I have had the most positive and absolute knowledge that the crossing of races does produce such a change in the jaw as to bring about a destruction of teeth, and irregularity of position in the arch. Now what should be the remedy? As in these ideal cases there is a single point of contact, every arch that can should be restored to the ideal type. When you get teeth out of all sorts of races, it sometimes becomes difficult to bring back this point of contact.

A man must weigh cases and decide for himself in each particular mouth how he shall proceed so as to bring it as near as possible to the highest type of ideal perfection. The formation of the teeth and their relation to each other should decide him as to the character of operation to be performed. It might require contouring or it might require cutting. I do not think anybody can safely lay down any precise rules in regard to this. I should always pursue the plan thatI thought would bring the mouth back to the nearest approach to a normal condition.

In regard to amalgams and plastic filling materials generally, I occupy very conservative ground. I have found all these fillings of some use somewhere, and I have used them wherever I thought it best. I would be as violently opposed to a man who would all together condemn cohesive gold, as I would to a man who would condemn non-cohesive foil. I have seen too many fillings that have been standing in the mouth for years and years of non-cohesive and cohesive gold; fillings which have stood the test of time. I think it is much easier to put in a filling of non-cohesive, than one of cohesive gold. I have seen fillings of soft foil that have been preserving the teeth for many years, that you could stick an excavator through from one end to the other. Carelessly put in, a filling of cohesive gold you will all admit will fail at once.

Dr. Coy. Mr. President, I have used amalgams for over thirty years. I have amalgam fillings in this city that have been inserted over a quarter of a century. They were made of Mexican coin filed up. If these amalgams leak, it is better that all leak. They have preserved the teeth. We all know that in the use of gutta percha fillings we preserve the teeth, and we all know that gutta percha leaks. Is it absolutely necessary under all circumstances that moisture should be executed in order to prevent the teeth from decaying? I have found these amalgam fillings black, but doing good service in teeth that were too bad to fill with gold. If any gentlemen will tell me of anything that has yet been presented to the dental profession that will save the teeth twenty-five years, then I will use that instead of amalgam. The amalgams of the present day take a very fine finish, but the tendency to a globular form is very easily recognized. If you leave an exposed surface, oxydation will take place of course. In putting in an amalgam filling, I use a burnisher; I put some in and burnish it over, and then put some more in and burnish that over.

Dr. Duck. I would like to ask Dr. Coy if he came across teeth twenty-five years ago, like the teeth of the present day?

Dr. Coy. I would say that these teeth were nothing but shells.

Dr. Duck. You filled them at that time pretty much as you would now?

Dr. Coy. Yes, sir, I did.

The President announced clinics for the following morning, by Drs. W. W. Evans and C. E. Duck.

---

Baltimore, Thursday, Sept. 4th, 1879.

The Association met at 11 A. M., the President in the chair. The reading of the minutes was dispensed with.

Dr. D. C. Ireland was elected an honorary member of the Association, and Dr. W. H. Hoopes an active member.

The Executive Committee made a report on Instruments and Appliances, which, on motion, was recommitted and a modified report made as follows:

Upon Instruments and Appliances your Committee would report that they are much pleased with the nickel-plated, socket handle instruments of Johnston Bros., and cheerfully recommend them to general use. The advantages are that they are a cheap article, and that the difference in the patterns of the handles enables the operator to rapidly select the points he desires to use.

The hand piece of Johnston Bros. we cannot recommend. It is intricate in its construction, but the main objection is that but one form of instruments fits it.

In regard to the White Chair, we have no hesitation, and are unanimous in the .opinion that it is the best chair that has been presented to the profession up to this time, and that it is a luxury to both patient and operator.

We also think very highly of, and call attention to the apparatus exhibited by Dr. T. S. Waters, of Baltimore, for holding engine burs, keeping sufficiently oiled to protect them from oxidation, and the addendum of an arrangement for heating water, with alcohol lamp in combination with it.

R. B. Winder,

B. F. Coy,

H. C. Thompson.

*Executive Committee.*

Under Miscellaneous Business, Dr. Foster offered the following resolution which was adopted:

*Resolved*, That when any new material or compound for filling is offered to any member of this Association, that member be requested to refer the matter to the section on Metallurgy and Chemistry of the Metals, and that section be requested to furnish the members with an opinion as to its merits.

Dr. Thompson moved that all other new materials coming before the profession be referred to appropriate sections. Adopted.

The President announced the discussion of papers on Operative Dentistry and Metallurgy, open for discussion.

Dr. Coy stated that he wished to amend his report on Operative Dentistry, by speaking of conservation of pulps and roots of teeth.

Dr. Atkinson made some appropriate remarks on the subject.

On motion of Dr. Coy, these sections were passed.

Section 9 (Artificial Dentistry,) was also passed.

Dr. Noble presented from the Section on Dental Education and Literature, the following report:

It is felt that this section has not been conducted upon the plan, or with the vigor necessary to the production of the best results, but the blame does not all lay with the chairman. It is an old, well-worn subject, yet it is the foundation of all our work, and upon the right and proper education of those entering our profession, depends our future standing.

It is, we believe, now conceded that the only proper entrance to our profession is through a regular course of study in a dental college, and the holding of a diploma which should be D. D. S., showing that the holder has passed a satisfactory examination in dentistry; many of our societies require a diploma to be eligible to membership—we hope it soon will be in all.

In last year's report the hope was expressed that the two colleges in our jurisdiction should unite or consolidate, but we did not expect to see it accomplished within the year, yet such is the pleasing fact, and we now have but one dental college, the old Baltimore College of Dental Surgery, in which building we are now assembled, founded mainly by the labor and energy of Chapin A. Harris, whose very name makes our hearts swell with pride, and whose labors are a rich heritage to our profession. It is generally felt, that by the consolidation of the two schools and the unity of forces, a better school will be the result. The College Board of Visitors comprise some of our oldest and most skillful operators, and we feel sure that they will examine carefully all students who present themselves for the honors of the college, so that no unworthy person shall receive its diploma. We think if the confering of degrees were placed in the hands of a board of regents, as in the plan of the Maryland Dental College, it would be likely to elevate the standard of graduation.

We would reiterate last year's report in reference to the appointment of teachers, that the voice of the alumni should be consulted by nomination, or some plan that would give the profession a voice in the selection; and also that each professor be elected for a definite term, so that the chaff could be winnowed out.

The length of pupilage and the time one shall be in attendance upon lectures before he shall be entitled to go forward for examination, was brought forward and discussed at the last meeting of the American Dental Association, and the attempt made to fix the minimum limit of attendance to two sessions. While we think that no one can properly be instructed in a less time than two years, yet to say a proper knowledge of dentistry can only be obtained by an attendance of two years without reference to the knowledge he may have obtained elsewhere, would be very unjust to any one who had spent years in study and practice outside of college walls. Thorough preparation should be insisted upon without reference to the time or place of its acquirement, and no diploma should be granted under 21 years, and only upon the most thorough and strict examination. No student or pupil should be received without a careful preliminary examination as to his education, and moral as well as mental standing. It is feared that these qualifications have in many cases been disregarded both in colleges and private offices.

Clinical instruction should be more carefully attended to, so as to give the student all the different ways of operating, and the more the better; yet a competent instructor should always be at hand to direct and explain. My observation in different schools last winter, showed a great need of a competent head to direct and lead students. They were left too much to their own resources rather than shown how to perform any given operation. There must be thoroughness and ability in the operating room and laboratory, as well as in the lecture-room, for all operations must be good, or our knowledge will be as "sounding brass." There must be aptness in teaching, and judgment to know what is in a student; and honesty and courage to reject all who do not come up to a proper standard.

In most of our colleges there is a great lack of cleanliness; that is a bad example to the student; there ought to be a greater abundance of napkins and towels, and the use of them enforced by precept and example, and the student be taught to be neat and cleanly in all his ways. Our college is in need of a better building and more extended accommodations. I understand that the State has helped our brethren of the medical profession. Can we not hope for the same? It is the oldest; let us labor to make it the best in the country; and it can be so by a little judicious pruning and grafting, and personal jealousies be not allowed to mar its usefulness.

---

### ARTICLE II.

[Continued from last Number.]

